# Microbiome Analysis Investigating the Impacts of Fermented Spent Mushroom Substrates on the Composition of Microbiota in Weaned Piglets Hindgut

**DOI:** 10.3389/fvets.2020.584243

**Published:** 2020-11-11

**Authors:** Qien Qi, Qiaoli Peng, Min Tang, Dongling Chen, Huihua Zhang

**Affiliations:** ^1^School of Life Science and Engineering, Foshan University, Foshan, China; ^2^Guangdong Province Key Laboratory of Animal Nutritional Control, College of Animal Science, South China Agricultural University, Guangzhou, China; ^3^Guangdong Yihao Foodstuff Co., Ltd., Zhanjiang, China

**Keywords:** fermented spent mushroom substrates, weaned piglets, intestinal health, microbiota, immunity

## Abstract

The purpose of this study was to investigate the effects of fermented spent mushroom substrates (FSMS) on growth performance, serum biochemical, gut digestive enzyme activity, microbial community, genes expression of tight junction proteins, and volatile fatty acids in the hindgut (colon and cecum) of weaned piglets. A total of 100 weaned Yihao native pigs (native × Duroc, 50 males and 50 females) were allocated to two groups with five replicates and 10 pigs per replicate. Pigs in the control group were fed a basal diet (BD group), and the others were fed basal diets supplemented with 3% FSMS (FSMS group). Relative to the BD group, it had better results for final weight, average daily gain, and feed conversion ratio in the FSMS group but not significant (*p* > 0.05), which was accompanied by improved serum triiodothyronine, immunoglobulin G, and immunoglobulin A (*p* < 0.05) but lower serum total protein, albumin, total cholesterol, and total triglyceride during the overall period (*p* < 0.05). Similarly, FSMS significantly upregulated (*p* < 0.05) the messenger RNA expression of duodenal tight junction proteins such as tight junction protein 1, tight junction protein 2, and occludin. Meanwhile, isobutyric acid, valeric acid, and isovaleric acid levels were increased, whereas propanoic acid was decreased (*p* < 0.05) in the FSMS group than the BD group. In addition, the piglets in the FSMS group changed the microbial diversity in the colon and cecum. 16S rRNA gene sequencing-based compositional analysis of the colonic and cecal microbiota showed differences in the relative abundance of bacterial phyla (*Firmicutes, Bacteroidetes*, etc.), genus (*Lactobacillus, Streptococcus, Roseburia*, etc.), and species (*Lactobacillus gasseri, Clostridium disporicum*, etc.) between the BD and FSMS fed piglets. In conclusion, dietary supplementation with FSMS benefited to the intestinal mucosal barrier, immunity, and composition of the microbiota.

## Introduction

Early weaning is a widespread practice in modern settings of pig production. At that time, piglets are exposed to a variety of stressors, including abrupt separation from sow and changes in diet and environment, which jointly result in a period of transient anorexia, gut mucosal atrophy, changes in intestinal microbiota composition, and weakening of immune system (1–3). Due to lack of a fully functional immune system, before, 5–6-week-old and weaned piglets are highly susceptible to multitudinous stressors leading to increase pathogenic bacteria and affecting intestinal health even death (4–6).

Antibiotics have long been used to solve problems in the weaning period and to promote the growth and health of piglets (7). Nonetheless, their indiscriminate use and misuse have led to antibiotic resistance and are potentially dangerous to human health (8, 9). In this scenario, there were reports that probiotics can be used as a substitute to in-feed antibiotics (10).

Probiotic defined as “live micro-organisms which when administered in adequate amounts confer a health benefit on the host” has gained much attention in recent years. It helps to relieve weaning stress, regulate intestinal flora, moreover, reduce diarrhea, etc. (8, 11). The *Bacillus* genus, a spore-forming bacterium, appears to be more resistant to adverse environments than most others and often used as porcine probiotic (12). It has been reported to reduce the number of intestinal toxigenic *Escherichia coli*, the incidence of diarrhea, and the mortality of weaned piglets and increase the immune effect (13). In addition, an effective probiotics administration is microbial fermentation of the feed (14).

Mushrooms have been used as food or medicine for thousands of years. Several bioactive constituents have been isolated from mushrooms, including small molecule compounds, polysaccharides, proteins, polysaccharide–protein complexes, etc. (15–17). Bioactive proteins include lectins, fungal immunomodulatory proteins, ribosome-inactivating proteins, antimicrobial proteins, ribonucleases, and laccases, which have attracted widespread attention of scientists due to their pharmaceutical potential (18–20). At present, many reports have proposed mushrooms as a feed ingredient directly to the animal diet or a small amount of it as a functional additive (21, 22). However, there are few reports in which the mushrooms is fermented firstly and then used as the main ingredient of the diets of livestock and poultry.

Fermented spent mushroom substrates (FSMS) is a large by-product of local food processing, produced by *Bacillus subtilis* fermentation of mushrooms, which is rich in mushroom small peptide, microbial bacteria, and their metabolites. FSMS was added to the diet of weaned piglets to explore its effects on growth performance, serum biochemical parameters, digestive enzyme activity, volatile fatty acids (VFAs), and microbial community. In addition, the messenger RNA (mRNA) expression of tight junction protein 1 (TJP1), tight junction protein 2 (TJP2), and occludin (OCLN) in the duodenum was evaluated to study the effects of FSMS on intestinal mucosal permeability of piglets.

## Materials and Methods

### Animal Ethics Statement

The experimental proposals and procedures for the care and treatment of the pigs were approved by the Animal Care and Use Committee of Foshan University, which were in accordance with ethical standards in Laboratory Animal—Guideline for ethical review of animal welfare (The National Standard of the People's Republic of China GB/T 35892-2018).

### Animals

One hundred weaned Yihao native pigs (native × Duroc) with the same genetic background, the same batch, and normal growth and development (males and females, weaned on 26 days of age, 4.10 ± 0.44 kg) were randomly assigned to two groups with five replicates and 10 pigs per replicate. Pigs in the control group were fed a basal diet (BD group), and the others were fed basal diets supplemented with 3% FSMS (FSMS group). All piglets were in a closed pigpen, the temperature was maintained at 25–30°C, and relative humidity was between 65 and 75% during the experimental period. The diets and water were provided with *ad libitum* throughout the 33-day feeding trial. At the same time, the pig house and trough were cleaned on time every day. Management and prevention of the epidemic were carried out in accordance.

### Diets

The experimental diets were formulated to meet or exceed the nutrient recommendations for lean-fat type growing–finishing pig in China (Table 1) (*NY*/T65, 2004). An experimental diet (FSMS) was formulated by adding 3% FSMS into the control diet. Diets BD and FSMS were identical with respect to all nutrients with restrictions in the linear programming formulation, thus providing equal amounts of the most important nutrients.

**Table 1:** Composition and nutrient levels of the diets (%, as-fed basis).

**Items**	**Diet**	
	**BD**	**FSMS**
**Ingredients (%)**		
Fermented *Lentinula edodes* residue		3.00
Corn (for suckling pigs)	38.00	36.86
Extruded corn	24.00	23.28
Whey powder	10.00	9.70
Soybean meal	11.00	10.67
Fermented soybean meal	9.00	8.73
Fish meal	4.00	3.88
premix	4.00	3.88
**Calculated nutrient content**		
DE (kcal/kg)	3,403.18	3,401.46
Crude protein (%)	18.91	18.90
Crude fiber (%)	1.98	2.16
Lysine (%)	1.03	1.00
Methionine (%)	0.34	0.33
M + C (%)	0.65	0.63
Threonine (%)	0.74	0.72
Tryptophan (%)	0.22	0.21

*Provided per kilogram of complete diet: vitamin A, 25,000 IU; vitamin D3, 75,000 IU; vitamin E, 350 mg; vitamin K3, 150 mg; niacin, 700 mg; D-pantothenate, 520 mg; vitamin B2, 170 mg; vitamin B6, 100 mg; vitamin B12, 1 mg; folic acid, 170 mg; D-biotin, 1.7 mg; choline chloride, 400 mg; Mn (MnSO_4_.H2O), 7.5 g; Fe (FeSO_4_.H2O), 10 g; Zn (ZnSO_4_.H2O), 75 g; Cu (CuSO_4_.5H2O), 8 g; I (KI), 50 mg; Se (Na_2_SeO_3_), 30 mg*.

*Nutritional components of fermented spent mushroom substrates (FSMS): estimated DE, 3,346 kcal/kg; crude protein, 18.5%; crude fiber, 8%; small peptide, 8.93%; lysine, 0.66%; methionine, 0.2%; threonine, 0.62%; tryptophan, 0.73%; valine, 0.96%; leucine, 1.12%*.

### Measurement of Growth Performance Index

The feed consumption per pen was recorded every day to calculate the average daily feed intake. The body weight (BW) of all pigs was recorded at the beginning and the end of the study period to determine average daily gain (ADG).

### Determination of Serum Biochemical Parameters

At the end of the experiment, six pigs from each group (*n* = 6 barrows, based on the average BW in each pen) were selected and then sampled. After fasting for approximately 12 h, blood samples were collected by cardiac puncture, and sera were isolated by centrifugation at 3,000 rpm for 10 min. The levels of immunoglobulin (Ig) proteins (IgG, IgA), thyroxine (T3, T4), and insulin-like growth factor (insulin-like growth factor 1) were determined using commercially available swine enzyme-linked immunosorbent assay kits from Shanghai Enzyme-linked Biotechnology Co., Ltd, China. Total protein (TP), albumin (ALB), total cholesterol (TC), triglyceride (TG), and urea (blood urea nitrogen) kits were purchased from Nanjing Jiancheng Bioengineering Research Institute, China.

### Determination of Digestive Enzyme Activity

After fasting for ~12 h, the pigs were euthanized by electrical stunning and exsanguination. The digesta of the duodenum, jejunum, ileum, cecum, and colon were collected and homogenized. Digestive enzyme activities (trypsin, lipase, α-amylase, and β-amylase) of the contents from the duodenum were measured by using appropriate kits (Jiancheng Bioengineering, Nanjing, China).

### Expression of Genes Related to Intestinal Permeability

Scraping mucosa was scraped with a cutter blade, transferred to a 2-ml centrifuge tube, and immersed in 1-ml RNA later. Total mRNAs were extracted from the mucosa by traditional Trizol method and then reversely transcribed to complementary DNAs according to the manufacturer's instructions with HiScript® II Q RT SuperMix for quantitative polymerase chain reaction (PCR) (+gDNA wiper) from Vazyme Biotech Co., Ltd., China.

Real-time reverse transcription PCR was carried out using a Power SYBR® Green PCR Master Mix (Promega, American) with a QuantStudio 3 Real-Time PCR System (Applied Biosystems; Thermo Fisher Scientific, Inc.). The PCR cycling condition was as follows: one cycle at 95°C for 5 min, then 40 cycles of 95°C for 15 s, 58/59°C for 40 s, 72°C for 15 s, and melt curve at 58/59°C for 1 min, 95°C for 15 s. For each sample, reactions were duplicated, and the average mRNA expression level of each gene was normalized to the reference gene β-actin level and determined using the 2^−ΔΔCt^ method. The primer pairs for measuring the levels of each gene are listed in Supplementary Table 1.

### Determination of Volatile Fatty Acids

For determination of VFAs, according to steps described in *Effect of tea saponin on rumen fermentation in vitro* (23), 10 g of colon digesta was placed in centrifugal tubes, mixed uniformly with 10 ml of 25% ortho-phosphoric acid and then centrifuged at 10,000 rpm for 10 min. The supernatant was decanted into another test tube, capped, and stored in a refrigerator at 4°C until analyzed using gas chromatography (SP-3420, Beijing Analysis Instrument Factory). The supernatant was filtered through a 0.22-μm membrane and then injected into a 2 × 6-mm glass column packed with Chromosorb (80–100 mesh). The temperature of the injector/detector and the column were 260 and 220°C, respectively. Nitrogen was used as a carrier.

### Microbial Community

Fecal samples from cecum and colon were collected and transferred to a 2-ml centrifuge tube and immersed in 1-ml RNA later. Microbial DNA extraction was performed using a QIAamp DNA Stool Mini Kit (QIAGEN, Germany) according to manufacturer's protocol. DNA concentrations of every sample were quantified using a Nanodrop 2000 spectrophotometer (Thermo Fisher Scientific, Wilmington, DE, United States). The genes of all bacterial 16S rRNA in the region of V3–V4 were amplified by PCR using a universal forward primer 338F (5′-ACTCCTRCGGGAGGCAGCAG-3′) and a reverse primer 806R (5′-GGACTACCVGGGTATCTAAT-3′) (24). PCR reactions were carried out in 30-μl reactions with 15 μl of Phusion High-Fidelity PCR Master Mix (New England Biolabs), 0.2 μM of forward and reverse primers, and 10-ng template DNA. Thermal cycling consisted of initial denaturation at 98°C for 1 min, followed by 30 cycles of denaturation at 98°C for 10 s, annealing at 50°C for 30 s, and elongation at 72°C for 30 s, finally 72°C for 5 min.

The authors mixed the same volume of 1 × loading buffer (contained SYB green) with PCR products and operate electrophoresis on 2% agarose gel for detection. PCR products were mixed in equidensity ratios. Then, mixture PCR products were purified with the Qiagen Gel Extraction Kit (Qiagen, Germany). Sequencing libraries were generated using Illumina TruSeq DNA PCR-Free Library Preparation Kit (Illumina, USA) following the manufacturer's recommendations, and index codes were added. The sequences were performed by Illumina Hiseq platform (NovoGene Ltd and Biomarker Ltd., respectively).

The QIIME (version 1.9.1, http://qiime.org/scripts/split_libraries_fastq.html) software package was used to demultiplex and quality-filter raw sequence data generated from 16S rRNA MiSeq sequencing (25). Gaps in each sequence were discarded from all the samples to decrease the noise generated by the screening, filtering, and pre-clustering processes, as described previously (26). Operational taxonomic units (OTUs) were clustered as a similarity cutoff of 97% using UPARSE (version 7.0.1001, http://drive5.com/uparse/), and unnormal gene sequences were identified and deleted using UCHIME (27). With each OTU, the representative sequence was analyzed using the Ribosomal Database Project classifier (RRID: SCR_006633) against the Silva (http://www.arb-silva.de/) 16S rRNA database using a confidence level of 90%.

The bacterial diversity, such as rarefaction analysis, the number of observed OTUs, coverage abundance estimator, richness estimator (Chao 1 and ACE), and diversity indices (Shannon and Simpson), were calculated using MOTHUR software (version 1.35.12) according to previous instructions (28).

### Statistics

Where appropriate, results were analyzed using an unpaired, two-tailed Student's *t*-test (*TTEST* function in Microsoft Excel 2007) to calculate a *p*-value. Values <0.05 were deemed statistically significant.

## Results

### Growth Performances

The influences of adding FSMS on pig growth performance are shown in Table 2. There are 50 piglets in each group, and no significant differences were noted for the initial weight. In addition, compared with the BD group, there were no significant differences between the two groups for the final weight, ADG, and FCR in the FSMS group over the entire feeding period (*p* > 0.05).

**Table 2:** Influence of FSMS addition on porcine growth performance indexes.

**Items**	**BD**	**FSMS**	***p*-value**
Sample	50	50	
Initial weight (kg)	4.15 ± 0.45	4.05 ± 0.43	0.898
Final weight (kg)	14.69 ± 1.29	15.08 ± 1.06	0.836
ADG (g)	301.28 ± 24.86	315.00 ± 18.01	0.816
FCR	1.33 ± 0.02	1.29 ± 0.01	0.092

### Serum Biochemical Parameters

The influences of FSMS addition on porcine blood biochemical indexes are shown in Table 3. IgG, IgA, and T3 were all significantly higher in the FSMS group (*p* < 0.05). In parallel with this, the content of TP, ALB, TC, and TG in the FSMS group were significantly decreased (*p* < 0.05). However, there was no significant difference between the two groups for the T4, insulin-like growth factor 1, and blood urea nitrogen over the entire feeding period (*p* > 0.05).

**Table 3:** Influence of FSMS addition on porcine blood biochemical indexes.

**Items**	**BD**	**FSMS**	***p-*value**
IgG (mg/ml)	21.4 ± 2.86^b^	32.23 ± 3.39^a^	0.001
IgA (ug/ml)	679.06 ± 140.27^b^	866.00 ± 50.21^a^	0.026
T3 (pmol/L)	11.52 ± 1.94^b^	14.73 ± 1.69^a^	0.017
T4 (pmol/L)	45.71 ± 4.98	50.47 ± 2.71	0.108
IGF-1 (ng/ml)	595.19 ± 72.79	754.35 ± 148.68	0.103
TP (mg/ml)	66.39 ± 10.05^a^	49.61 ± 7.43^b^	0.017
ALB (g/L)	30.13 ± 7.12^a^	21.06 ± 1.94^b^	0.025
TC (mmol/L)	7.69 ± 1.89^a^	5.46 ± 0.87^b^	0.049
TG (mmol/L)	4.37 ± 2.17^a^	1.62 ± 0.37^b^	0.023
BUN (mmol/L)	4.03 ± 0.45	3.73 ± 0.73	0.470

*IgG, immunoglobulin G; IgA, immunoglobulin A; T3, triiodothyronine; T4, tetraiodothyronine; IGF-1, insulin-like growth factor 1; TP, total protein; ALB, albumin; TC, total cholesterol; TG, triglyceride; BUN, blood urea nitrogen. Values are means ± SEM (n = 6). Results were analyzed by t-test, and the variant letter in the same row indicated a significant difference when P < 0.05*.

### Digestive Performance

The influences of FSMS addition on porcine duodenum content digestive enzyme activities are shown in Table 4. Over the entire 33-day growth trial, the trypsin, lipase, α-amylase, and β-amylase of piglets in the FSMS group had no significant difference from the BD group.

**Table 4:** Influence of FSMS addition on porcine duodenum content digestive enzyme activities.

**Items**	**BD**	**FSMS**	***p-*value**
Trypsin (U/mg)	12.71 ± 8.05	46.32 ± 37.89	0.200
Lipase (U/g)	203.31 ± 203.59	214.87 ± 45.03	0.915
α-Amylase (mmol/L)	6264.89 ± 389.63	5846.31 ± 641.09	0.328
β-Amylase (mmol/L)	79.06 ± 11.89	79.81 ± 5.32	0.901

*Values are means ± SEM (n = 6). Results were analyzed by the t-test, and the variant letter in the same row indicated a significant difference when P < 0.05*.

### Gene Expression of Tight Junction Proteins

**Figure 1:**
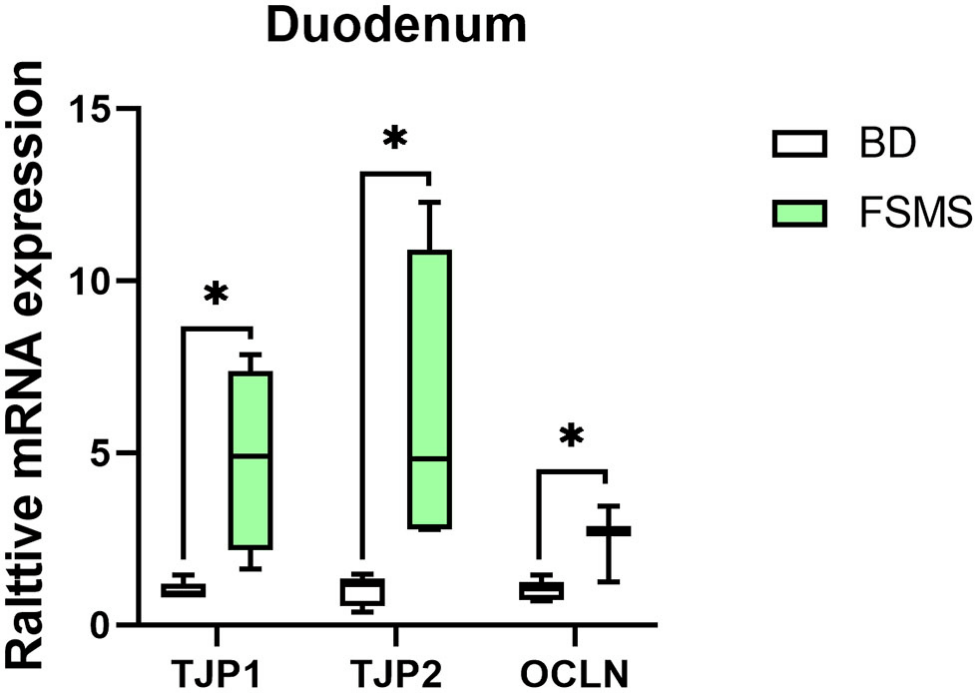
Influence of FSMS addition on the tight junction transmembrane protein, tight junction protein 1 (TJP1), tight junction protein 2 (TJP2), and occludin (OCLN) mRNA expression. *p < 0.05, **p < 0.01.

The expression of genes related to intestinal permeability in the duodenum is shown in Figure 1. According to the results, the addition of FSMS in the diet of weaned piglet significantly upregulated the expression of TJP1, TJP2, and OCLN genes in the duodenum (*P* < 0.05).

### Volatile Fatty Acids

The effects of different dietary treatments on colonic content VFA are as presented in Table 5. The piglets in the FSMS group showed a lower propanoic acid than those in the BD group (*P* < 0.05). The contents of isobutyric acid, valeric acid, and isovaleric acid in the FSMS group were significantly higher than those in the BD group (*P* < 0.05). Additionally, there was no significant difference in the content of acetic acid and butyric acid between the two groups (*P* > 0.05).

**Table 5:** Influence of FSMS addition on porcine colon digesta volatility fatty acids (VFAs) production.

**Items**	**BD**	**FSMS**	***p-*value**
Acetic acid (mmol/L)	59.59 ± 5.78	50.93 ± 11.03	0.170
Propanoic acid (mmol/L)	26.16 ± 1.74^a^	19.38 ± 6.55^b^	0.036
Butyric acid (mmol/L)	5.99 ± 0.84	4.37 ± 1.28	0.056
Isobutyric acid (mmol/L)	0.38 ± 0.17^b^	1.95 ± 0.33^a^	0.000
Valeric acid (mmol/L)	0.78 ± 0.12^b^	1.34 ± 0.53^a^	0.050
Isovaleric acid (mmol/L)	0.62 ± 0.13^b^	2.58 ± 0.50^a^	0.000

*Values are means ± SEM (n = 6). Results were analyzed by the t-test, and the variant letter in the same row indicated a significant difference when P < 0.05*.

### Microbial Community

To evaluate the impact of FSMS addition on the microbial composition of cecal and colonic digesta, a total of 1,463,611 V3–V4 16S rRNA effective sequences from the 24 samples (12 cecal digesta samples and 12 colonic digesta samples), with an average of 60,984 sequences per sample, were used for subsequent analysis.

The influences of FSMS addition on porcine cecal digesta bacterial community are shown in Supplementary Table 2. Diversity indices and richness were analyzed to compare the differences in alpha diversity of microorganisms in cecum between the FSMS group and the BD group. Piglets in the FSMS group had higher diversity indices compared with those in the BD group, as reflected by the Shannon index with statistical differences (*p* < 0.05) but without affecting OUT numbers and Simpson index (*p* > 0.05). Curiously, for observed species, Chao1 and ACE, the BD group is significantly higher than the FSMS group (*p* < 0.05).

The influences of FSMS addition on porcine colonic digesta bacterial community are shown in Supplementary Table 3. Piglets in the FSMS group had a higher observed species and Shannon indices compared with those in the BD group (*p* < 0.05). However, no differences in OUT numbers, Goods coverage, Chao1, ACE, and Simpson index between the two groups were observed (*p* > 0.05).

**Figure 2:**
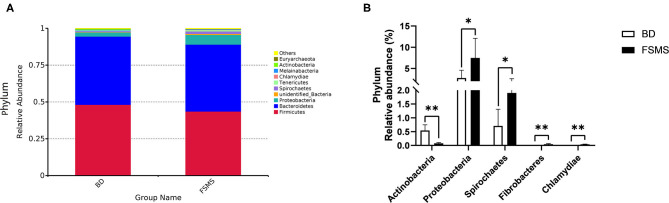
Relative abundance of bacterial phylum in the cecal digesta of pigs **(A)**. Significantly changed phyla in the cecal digesta **(B)**. Values were expressed as means ± SEM (*n* = 6). Statistical differences were calculated by Duncan: *p < 0.05, **p < 0.01.

The influences of FSMS addition on the porcine relative abundance of bacterial phylum in the cecal digesta are shown in Figure 2. A total of 10 different phyla were detected in the examined samples. The two groups showed very similar taxonomic compositions at the phylum-level distributions, and the major sequences obtained from the samples belonged to Firmicutes and Bacteroidetes, contributing 43.58 and 45.46% in the FSMS group and 48.20 and 46.36% in the BD group, respectively (Figure 2A). Piglets in the FSMS group demonstrated a significantly decreased (*P* < 0.05) abundance of bacteria belonging to the phyla Actinobacteria compared with the piglets in the BD group, whereas a significant increased (*P* < 0.05) abundance of Proteobacteria, Spirochaetes, Fibrobacteres, and Chlamydine compared with the pigs in the BD group (Figure 2B).

**Figure 3:**
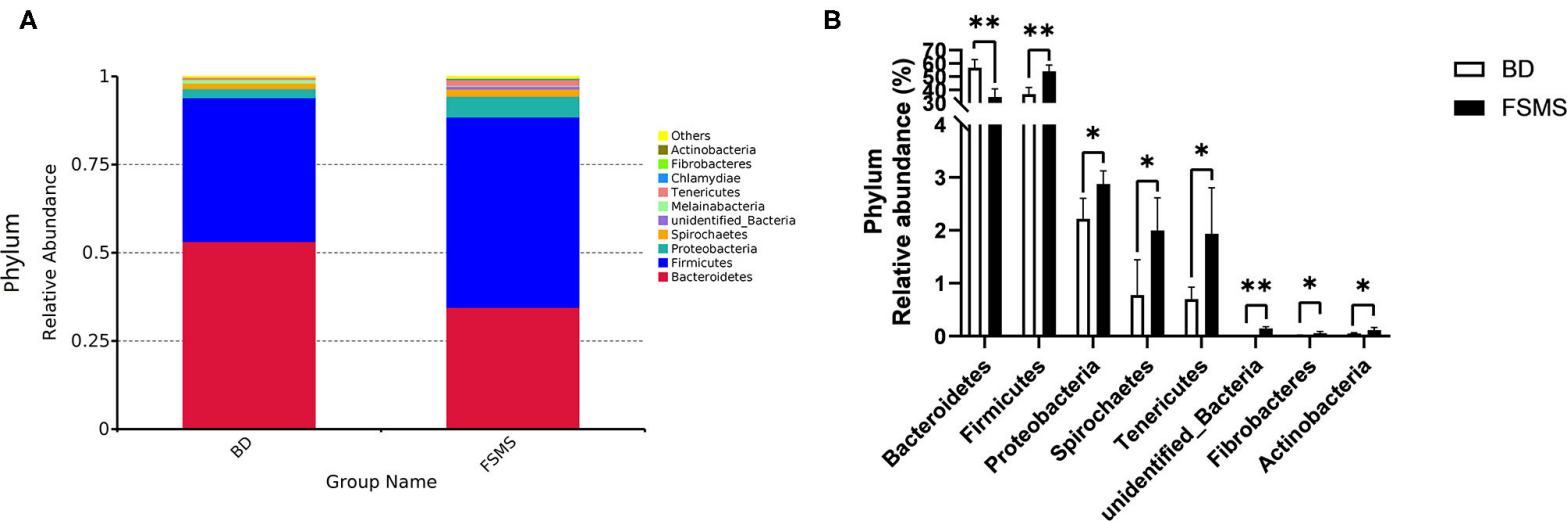
Relative abundance of bacterial phylum in the colonic digesta of pigs **(A)**. Significant changed phyla in the colonic digesta **(B)**. Values were expressed as means ± SEM (*n* = 6). Statistical differences were calculated by Duncan: *p < 0.05, **p < 0.01.

The influences of FSMS addition on the porcine relative abundance of bacterial phylum in the colonic digesta are shown in Figure 3. A total of 10 different phyla were detected in the examined samples. The two groups showed very similar taxonomic compositions at the phylum-level distributions, and the major sequences obtained from the samples belonged to Firmicutes and Bacteroidetes, contributing 54.02 and 34.52% in the FSMS group and 40.79 and 53.20% in the BD group, respectively (Figure 3A). Compared with the piglets in the BD group, piglets in the FSMS group demonstrated a significantly decreased (*P* < 0.01) abundance of bacteria belonging to the predominant phyla Bacteroidetes, whereas a significant increased (*P* < 0.05) abundance of Firmicutes, Proteobacteria, Spirochaetes, Tenericutes, unidentified bacteria, Fibrobacteres, and Actinobacteria compared with the pigs in the BD group (Figure 3B).

**Figure 4:**
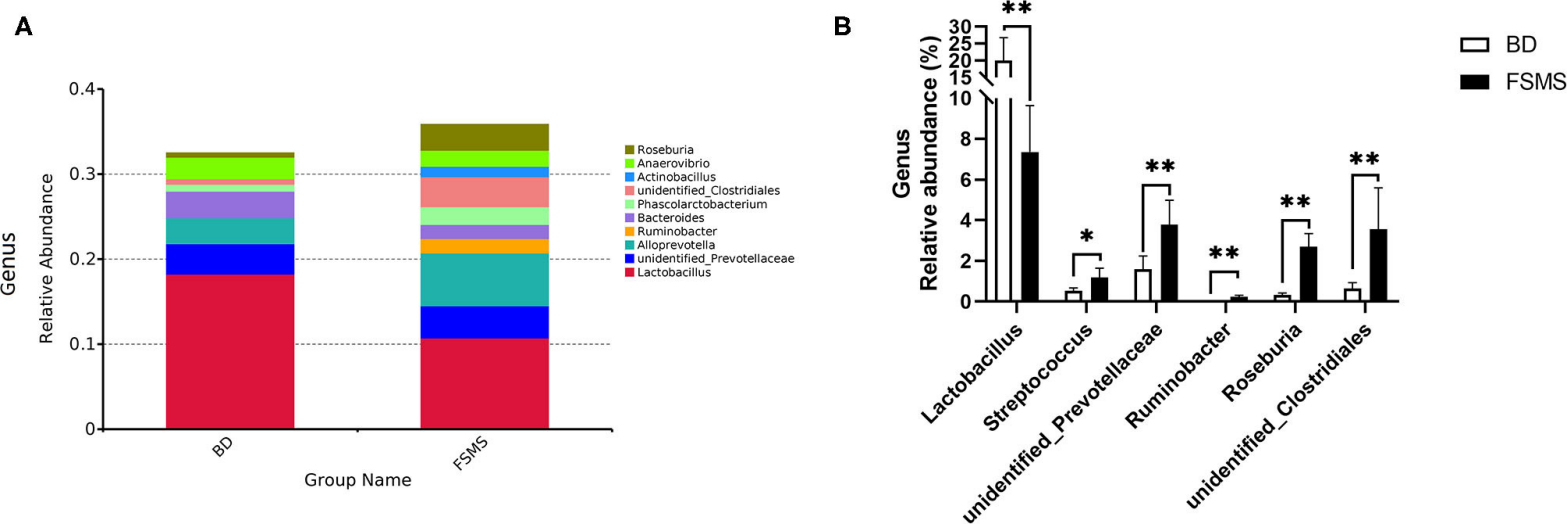
Relative abundance of bacterial genus in the cecal digesta of pigs **(A)**. Significant changed genera in the cecal digesta **(B)**. Values were expressed as means ± SEM (*n* = 6). Statistical differences were calculated by Duncan: *p < 0.05, **p < 0.01.

The influences of FSMS addition on the porcine relative abundance of the bacterial genus in the cecal digesta are shown in Figure 4. A total of 10 different phyla were detected in the examined samples. The two groups showed very similar taxonomic compositions at the genus-level distributions, but the major sequences obtained from the samples belonged to Lactobacillus and Alloprevotella in the FSMS group, contributing 10.73 and 6.20%, respectively, whereas the predominant genera were *Lactobacillus* and unidentified *Prevotellaceae* in the BD group, contributing 18.24 and 3.57%, respectively (Figure 4A). Compared with the piglets in the BD group, the piglets in the FSMS group demonstrated a significantly decreased (*P* < 0.01) abundance of bacteria belonging to the predominant genera *Lactobacillus*, whereas a significantly increased (*P* < 0.05) abundance of *Streptococcus*, unidentified *Prevotellaceae, Ruminobacter, Roseburia*, and unidentified *Clostridiales* (Figure 4B).

**Figure 5:**
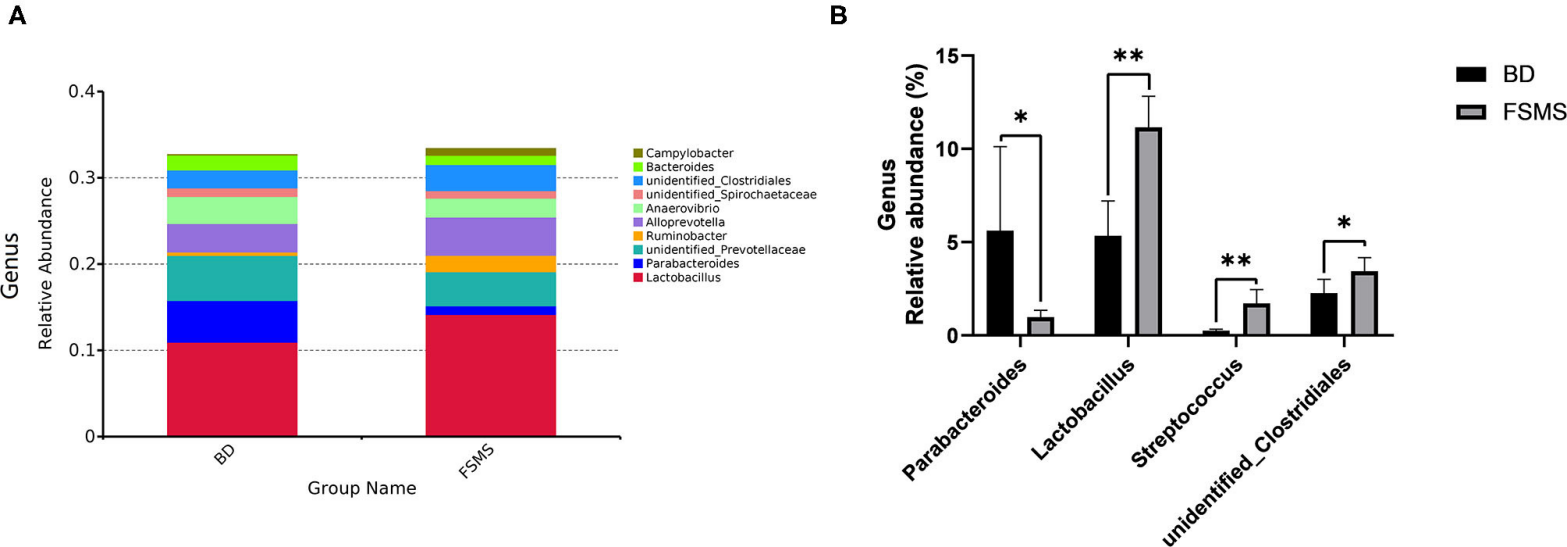
Relative abundance of bacterial genus in the colonic digesta of pigs **(A)**. Significant changed genera in the colonic digesta **(B)**. Values were expressed as means ± SEM (*n* = 6). Statistical differences were calculated by Duncan: *p < 0.05, **p < 0.01.

The influences of FSMS addition on the porcine relative abundance of the bacterial genus in the colonic digesta are shown in Figure 5. A total of 10 different phyla were detected in the examined samples. The two groups showed very similar taxonomic compositions at the genus-level distributions, but the major sequences obtained from the samples belonged to Lactobacillus and Alloprevotella in the FSMS group, contributing 14.17 and 4.43%, respectively, whereas the predominant genera were *Lactobacillus* and unidentified *Prevotellaceae* in the BD group, contributing 10.94 and 5.22%, respectively (Figure 5A). Compared with the piglets in the BD group, the piglets in the FSMS group demonstrated a significantly decreased (*P* < 0.01) abundance of bacteria belonging to the predominant genera *Parabacteroides*, whereas a significant increased (*P* < 0.05) abundance of Lactobacillus, Streptococcus, and unidentified Clostridiales (Figure 5B).

**Figure 6:**
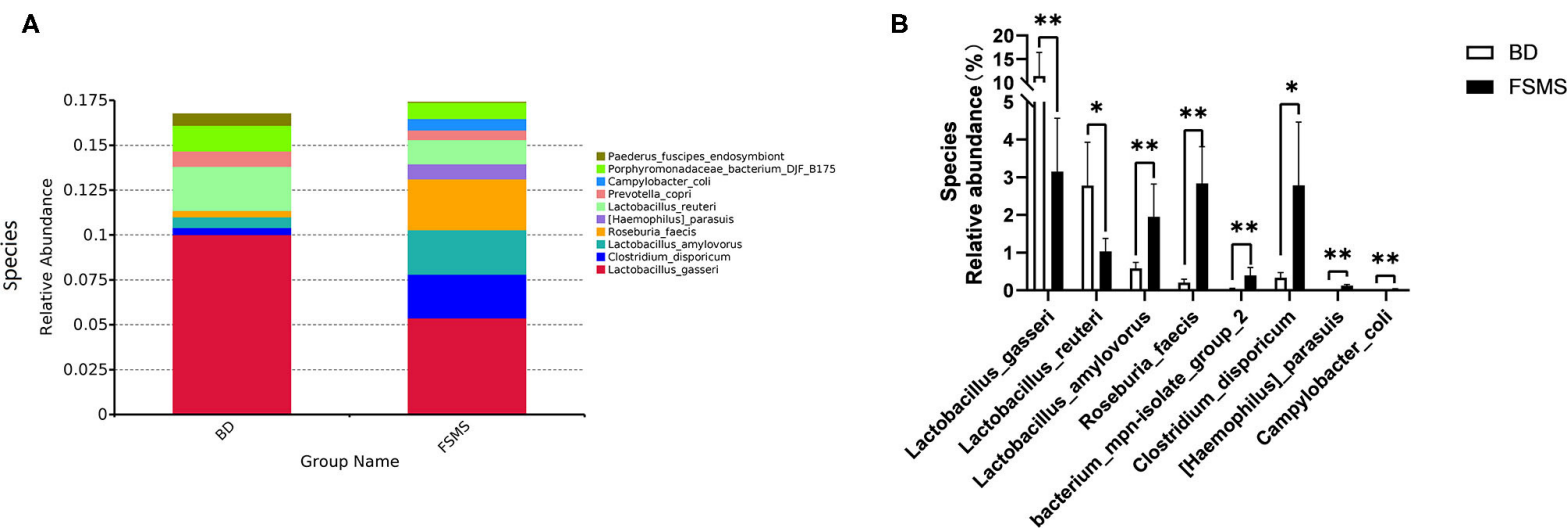
Relative abundance of bacterial species in the cecal digesta of pigs **(A)**. Significant changed species in the cecal digesta **(B)**. Values were expressed as means ± SEM (*n* = 6). Statistical differences were calculated by Duncan: *p < 0.05, **p < 0.01.

The influences of FSMS addition on the porcine relative abundance of bacterial species in the cecal digesta are shown in Figure 6. A total of 10 different species were detected in the examined samples. The two groups showed very similar taxonomic compositions at the species-level distributions, but the major sequences obtained from the samples belonged to *Lactobacillus gasseri* and *Roseburia faecis* in the FSMS group, contributing 5.38 and 2.84%, respectively, whereas the predominant genera were *L. gasseri* and unidentified *Prevotellaceae* in the BD group, contributing 10.02 and 2.46%, respectively (Figure 6A). Compared with the piglets in the BD group, the piglets in the FSMS group demonstrated a significantly decreased (*P* < 0.01) abundance of bacteria belonging to *L. gasseri* and *Lactobacillus reuteri*, whereas a significant increased (*P* < 0.05) abundance of *Lactobacillus amylovorus, R. faecis*, bacterium mpn-isolate group 2, *Clostridium disporicum*, (*Haemophilus*) *parasuis*, and *Campylobacter coli* (Figure 6B).

**Figure 7:**
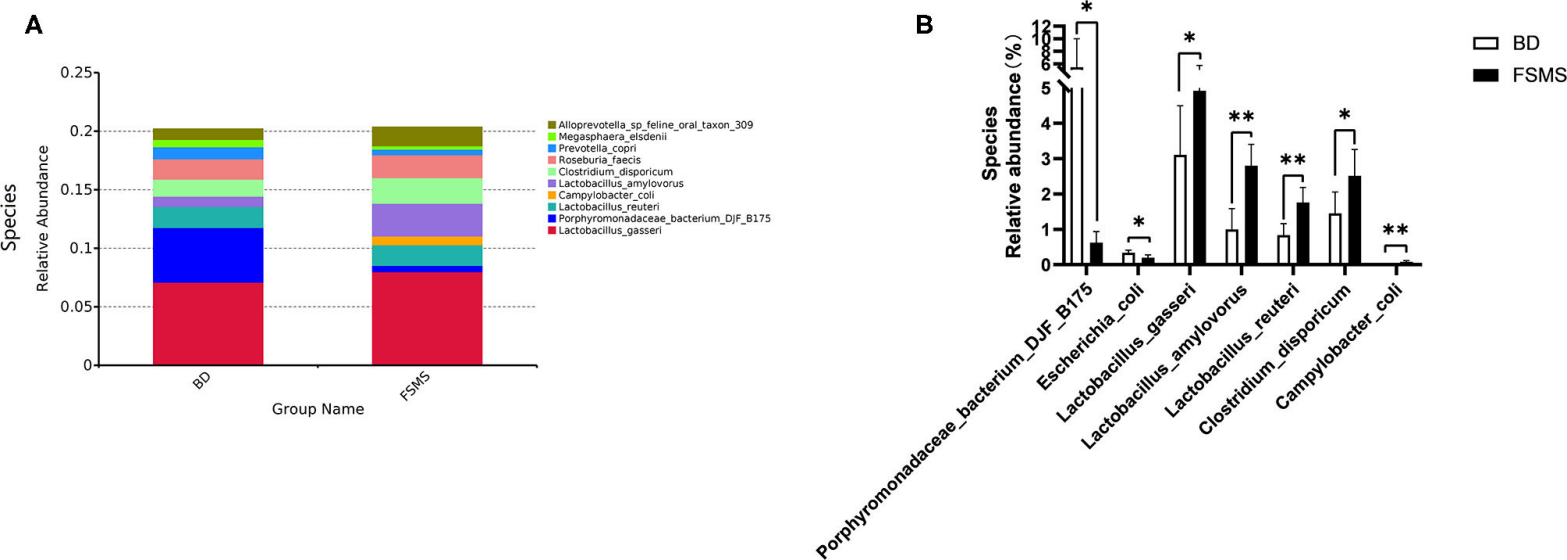
Relative abundance of bacterial species in the colonic digesta of pigs **(A)**. Significant changed species in the colonic digesta **(B)**. Values were expressed as means ± SEM (*n* = 6). Statistical differences were calculated by Duncan: *p < 0.05, **p < 0.01.

The influences of FSMS addition on the porcine relative abundance of bacterial species in the colonic digesta are shown in Figure 7. A total of 10 different species were detected in the examined samples. The two groups showed very similar taxonomic compositions at the species-level distributions, but the major sequences obtained from the samples belonged to *L. gasseri* and *L. amylovorus* in the FSMS group, contributing 7.99 and 2.80%, respectively, whereas the predominant genera were *L. gasseri* and *Porphyromonadaceae bacterium* DJF B175 in the BD group, contributing 7.09 and 4.68%, respectively (Figure 7A). Compared with the piglets in the BD group, the piglets in the FSMS group demonstrated a significantly decreased (*P* < 0.05) abundance of bacteria belonging to the *P. bacterium* DJF B175 and *E. coli*, whereas a significant increased (*P* < 0.05) abundance of *L. gasseri, L. amylovorus, L. reuteri, C. disporicum*, and *C. coli* (Figure 7B).

## Discussion

The intestinal barrier is vital for nutrient absorption and health in animals (29). However, various factors cause the piglets to stress and thus destroy the intestinal barrier during weaning (30). We do know antibiotics used to solve problems of piglets weaning but may lead to antibiotic resistance (31). It has been reported that probiotics can be an alternative to antibiotics and that fermented food is an ideal vehicle for the delivery of probiotics to animals. In addition, the fermented feed has antioxidant, immune, and antibacterial effects (32–35). Thus, in this study, we used FSMS to explore its effects on growth performance and intestinal health of weaned piglets.

### Growth Performances

Piglet growth performance is subjected to diet ingredients. Feeding fermented feed to piglets has given varying effects on growth performance. In the present study, the growth performance of piglets fed with FSMS additional feed was improved compared with that of piglets fed the same unadded diet. This type of results has also been observed with Hanwoo steers fed diet added microbially FSMS (36). The effect of microbially FSMS addition might be an increased intake of DM and CP effects. Additionally, the favorable impact could be due to the microbes. Hu et al. reported that the ADG and feed efficiency of feed weaned piglets with added bacillus increased (37). Huang et al. also reported that the dietary addition of bacillus increased the BW of the weaning piglets (38). Previous research has also indicated that fermented feed improved the flavor and palatability of feed as well as decomposing nutrients and reducing the wear and tear of the intestines, which was beneficial to the absorption and makes the piglets' performance improved (39, 40). It is believed that feeding FSMS favorably affected the growth of piglets.

### Serum Parameters and Tight Junction Proteins mRNAs Expression

Serum antibody levels are an indicator of humoral immunity, and an animal's systemic immune status may be reflected by the concentration of serum IgG (41). A previous study has documented that fermented *Ginkgo biloba* L. residues result in raised IgG and IgA (40). In this study, diet FSMS supplementation increased serum IgG and IgA of piglets by 50.05 and 27.53%, respectively. This beneficial effect might be due to the mRNA upregulation of TJP. TJPs play a positive role in improving body immune state (42). The function of the intestinal mucosal barrier is to prevent intestinal flora and toxins from entering the blood circulation, playing a crucial role in the health and growth of weaned piglets. Proper epithelial function mainly depends on tight junctions (43). The gastrointestinal tract is considered the largest immunological organ in the body, having a central role in regulating immune homeostasis (44). Therefore, duodenum TJP mRNA up-expression may be one of many reasons for the increase of serum Ig levels. This means that FSMS can be specifically applied to situations aimed to enhance animal immunity.

Piglet metabolism can be reflected by various biochemical indexes in serum. Protein is an important material basis for animal metabolism, growth, and development. The content of TP in blood demonstrates the metabolic activity of the substance (45, 46). ALB, synthesized and secreted by the liver, is generally regarded as protein synthesis and metabolic indicators related to piglet growth performance (47). Blood cholesterol and TG levels are linked to several cardiovascular diseases and are a major cause of atherosclerosis (48, 49). The concentration of TG and TC in the blood is an important index to measure the metabolism of blood lipids, reflecting the development of adipose tissue and the level of fat deposition. The results of our study, a decrease in TP, ALB, TC, and TG levels, may implicate that the supplementation of FSMS can reduce the burden of protein synthesis in the liver and regulate the fat deposition, meanwhile benefiting the cardiovascular system.

T3 is a major part of the physiological role of thyroxine in animals. It is said that thyroxine can also accelerate the transformation of cholesterol into cholic acid and cholate has the role of helping fat digestion, which can reduce the content of serum cholesterol. A previous study notes that when challenged with live yeast and superfine yeast in the basic diet, T3 of piglets significantly increased (*P* < 0.05). In our study, the concentrations of T3 increased by 27.86% and serum cholesterol decreased by 29.00% in the FSMS group. This was in agreement with the earlier discussed literature.

### Volatile Fatty Acids

The main place of pig microbial digestion is located in the back part of the digestive tract. Nutrients not absorbed by the stomach and small intestine enter the back intestine. After further fermentation by microorganisms, the fermentation products can be absorbed and enter the body to supply energy and regulate host metabolism and immunity. VFAs, including acetate, butyrate, propionate, isobutyric acid, valeric acid, and isovaleric acid, are produced mainly by microbial fermentation in the colon of mammals (50). As reported, valine and leucine were extensively metabolized within the rumen, yielding large quantities of isobutyric acids and isovaleric acid, respectively. Both amino acids tended to decrease the level of acetic acid, propanoic acid, and butyric acids (51, 52). In our study, FSMS was rich in valine (0.96%) and leucine (1.12%). This should be the cause of the variation of VFAs. In addition, propionate is synthesized via the succinate pathway, acrylate pathway, or propanediol pathway. This can be done by various microorganisms, including Bacteroidetes fermentation (53, 54). Our results showed that FSMS addition decreased the abundance of bacteria belonging to the predominant phyla Bacteroidetes in the colonic digesta. Therefore, the decrease of Bacteroidetes may also contribute to the decline in propionate.

### Microbial Community

There are trillions of microbes (including fungi, viruses, and bacteria) in the mammalian intestine harbors, which play an important role in maintaining intestinal environmental stability and host health, and they are considered as an additional organ (55). Microbiota influence intestinal barrier function (56). Changes in the species richness and diversity of bacteria help in the digestion and absorption of piglets (57). Changes in diet ingredients (such as carbohydrates, probiotics, etc.) affect the composition of intestinal microbes, thereby affecting intestinal health (58). In a well-balanced microbial environment, members of the following genera prevail: *Streptococcus, Lactobacillus, Bifidobacterium, Enterococcus, Eubacterium, Fusobacterium, Peptostreptococcus, Enterobacter, Bacteroides*, and *Porphyromona*, whereas the number of coliform bacteria *E. coli* and *Clostridium* sp. is lower (59).

In this study, 16S rRNA gene sequencing-based compositional analysis of the cecal and colonic microbiota showed differences in the relative abundance of bacterial phyla, genus, and species between the BD and FSMS-fed piglets, indicating that FSMS affected the gut microbiota. Ten bacterial phyla were identified in the cecum and colon, respectively, with Firmicutes, Bacteroidetes, and Proteobacteria dominating in the colon of the FSMS piglets, which was similar to findings found by Zhengjun Xie and Han (60). Among them, Firmicutes and Proteobacteria increased, but Bacteroidetes reduced significantly in the intestine. More abundance of Proteobacteria may be more favorable for animal health. A previous study demonstrates that a flora rich in Proteobacteria produces sepsis resistance in mice mediated by serum IgA (61). This corresponds to the findings that serum IgA increased in our study. Namely, the modulation of intestinal flora resulted in enhanced immunity. At the genus level, Lactobacillus, Streptococcus, and Roseburia were more abundant in the FSMS-fed piglets. Lactobacillus, Gram-positive bacteria is a normal flora of the gastrointestinal (GI) tract, and it can promote health in humans and other mammals (62). Studies have demonstrated that Lactobacillus is beneficial to maintain the intestinal epithelial barrier function in early-weaned piglets, and that may be used for preventing intestinal damage (63). In our study, *L. gasseri, L. amylovorus*, and *L. reuteri* species were all significantly increased in the colon. *Streptococcus* can produce organic acids and bacteriocins, thereby reducing intestinal pH, inhibiting the growth of pathogenic bacteria in the intestinal tract and benefit intestinal function (64). In addition, the enrichment of the genus *Streptococcus* in healthy infants has been reported by Zheng, and it revealed anti-inflammatory properties (65). *Roseburia*, SCFA producers, acidifies the intestinal environment, thus conducive to the growth of beneficial bacteria and inhibits the proliferation of harmful bacteria (66, 67). Like our study, *Roseburia* was also enriched in the 15% fermented Mao-tai lees group (68). Moreover, the abundance of *E. coli* decreased after feeding an FSMS diet. Therefore, the relative abundance of some beneficial bacteria increased (*Lactobacillus, Streptococcus*, and *Roseburia*) in the FSMS group indicated that feeding of the FSMS diet might have beneficial effects on the health of pigs.

## Conclusion

Three percent FSMS supplementation to piglets' diet can improve the composition of microbiota in the hindgut, intestinal barrier function, and overall immune status. This implies that FSMS can be used in animal diet formulation at an appropriate addition level. FSMS is a compound that contains mushroom small peptide, microbial bacteria, and their metabolites and which is the main effect component that needs to be confirmed.

## Data Availability Statement

The datasets generated for this study can be found in NCBI SRA, NCBI Accession No. PRJNA658850.

## Ethics Statement

The experimental proposals and procedures for the care and treatment of the pigs were approved by the Animal Care and Use Committee of Foshan University, which were in accordance with ethical standards in Laboratory animal—Guideline for ethical review of animal welfare (The National Standard of the People's Republic of China GB/T 35892-2018).

## Author Contributions

QQ and HZ conceived and designed the whole trial. MT and DC conducted the pig trial. QQ and QP conducted laboratory analyses. QQ, QP, and HZ wrote the manuscript. All authors contributed to the article and approved the submitted version.

## Conflict of Interest

MT and DC were employed by Guangdong Yihao Foodstuff Co., Ltd. The remaining authors declare that the research was conducted in the absence of any commercial or financial relationships that could be construed as a potential conflict of interest.
