# Supplementary material for: Microbiome Analysis Investigating the Impacts of Fermented Spent Mushroom Substrates on the Composition of Microbiota in Weaned Piglets Hindgut
Source: Front Vet Sci. 2020 Nov 11;7:584243. doi: 10.3389/fvets.2020.584243 (PMC7686581; doi:10.3389/fvets.2020.584243)
Supplement: Supplementary file 1 [file Data_Sheet_1.docx]

Microbiome analysis investigating the impacts of Fermented Spent Mushroom Substrates on the composition of microbiota in weaned piglets hindgut

*Qien Qi**^1,2＃^, Qiaoli Peng^1＃^, Min Tang^3^, Dongling Chen^3^, Huihua Zhang^1*^*

*1* *School of Life Science and Engineering, Foshan University, Foshan 528231, China*

*2 Guangdong Province Key Laboratory of Animal Nutritional Control, College of Animal Science, South China Agricultural University, Guangzhou 510642, China*

*3 Guangdong Yihao Foodstuff Co., Ltd., Zhanjiang 524000, China*

**Supplementary material**

**Summary**

The supporting information includes 3 supplementary tables.

**Table S1** Sequences of the primers for PCR

| Gene (GenBank accession No.) | Primer | Sequence (5′-3′) | PCR product size | Annealing temperature |
| --- | --- | --- | --- | --- |
| β-actin (KU672525.1) | F | GCATTGTCATGGACTCTGGG | 161 bp | 59 ℃ |
|  | R | CGTGGTGGTGAAGCTGTAGC |  |  |
| TJP1 (XM_003480423.4) | F | TGCCGCCTCCTGAGTTTGATA | 197 bp | 59 ℃ |
|  | R | CACCCCGCCGTTGCTGTTAAA |  |  |
| TJP2 (NM_001206404.1) | F | AAGATGCTGTTCTCTACCT | 152 bp | 58 ℃ |
|  | R | AGTTTCGTTCTCACATTC |  |  |
| OCLN (NM_001163647.2) | F | CTCAGCCAGCGTATTCTTTC | 281 bp | 58 ℃ |
|  | R | ATCTGTATAGCCCCCTCCAT |  |  |

**Table S2** Summary statistic of caecal digesta bacterial community

| Items | Diet | | P-value |
| --- | --- | --- | --- |
|  | BD | FSMS |  |
| OTU numbers | 61291 ± 2605 | 63095 ± 3796 | 0.406 |
| Observed species | 934.40 ± 68.85^a^ | 798.80 ± 30.11^b^ | 0.004 |
| Goods coverage, % | 99.67 ± 0.05^b^ | 99.77 ± 0.05^a^ | 0.007 |
| Richness |  |  |  |
| Chao1 | 1005.49 ± 96.29^a^ | 840.64 ± 54.30^b^ | 0.004 |
| ACE | 1018.11 ± 93.29^a^ | 852.68 ± 55.89^b^ | 0.004 |
| Diversity indices |  |  |  |
| Shannon | 6.48 ± 0.27^b^ | 7.11 ± 0.31^a^ | 0.009 |
| Simpson | 0.96 ± 0.01 | 0.98 ± 0.01 | 0.082 |

Values are means ± SEM (n = 6). Results were analyzed by one-way analysis of variance (ANOVA) with Turkey’s test, and the variant letter in the same row indicated significant difference when P < 0.05. OTU, operational taxonomic units; ACE, abundance-based coverage estimator.

**Table S3** Summary statistic of colonic digesta bacterial community

| Items | Diet | | P-value |
| --- | --- | --- | --- |
|  | BD | FSMS |  |
| OTU numbers | 63032 ± 2528 | 62808 ± 1915 | 0.871 |
| Observed species | 771.80 ± 56.45^b^ | 884.80 ± 15.77^a^ | 0.003 |
| Goods coverage, % | 99.73 ± 0.08 | 99.75 ± 0.05 | 0.687 |
| Richness |  |  |  |
| Chao1 | 828.29 ± 99.19 | 922.31 ± 50.91 | 0.072 |
| ACE | 836.17 ± 92.14 | 929.16 ± 57.97 | 0.071 |
| Diversity indices |  |  |  |
| Shannon | 6.84 ± 0.36^b^ | 7.41 ± 0.23^a^ | 0.018 |
| Simpson | 0.97 ± 0.03 | 0.98 ± 0.02 | 0.534 |

Values are means ± SEM (n = 6). Results were analyzed by one-way analysis of variance (ANOVA) with Turkey’s test, and the variant letter in the same row indicated significant difference when P < 0.05. OTU, operational taxonomic units; ACE, abundance-based coverage estimator.
